# Hospitals and medical specialization in Lisbon, 1880-1933

**DOI:** 10.1590/S0104-59702024000100052

**Published:** 2024-10-14

**Authors:** Isabel Amaral

**Affiliations:** i Associate Professor, Department of Applied Social Sciences/Interuniversity Center for the History of Science and Technology/NOVA School of Science and Technology. Caparica – Portugal; ima@fct.unl.pt

**Keywords:** History of hospitals, History of public health, Medical specialties, Portugal, Nineteenth and twentieth centuries, História dos hospitais, História da saúde pública, Especialidades médicas, Portugal, Séculos XIX e XX

## Abstract

This study analyzes the influence of hospitals on medical specialization and resulting implications for the history of public health from 1880 to 1933, with a focus on Lisbon as a case study. After the 1755 earthquake, a shift occurred from Renaissance-style hospitals to a network of hospitals that strategically occupied diverse urban spaces, significantly shaping the modernization of medicine and governmental healthcare policies. Employing the framework of the history of science, technology and medicine studies, this research enriches the historiography of hospitals, offering comprehensive insight into their societal significance during the study period.

## Resumo

O estudo analisa a influência dos hospitais na especialização médica e as implicações resultantes para a história da saúde pública de 1880 a 1933, com foco em Lisboa como um estudo de caso. Após o terremoto de 1755, ocorreu uma mudança dos hospitais de estilo renascentista para uma rede de hospitais que ocupava estrategicamente diversos espaços urbanos, moldando significativamente a modernização da medicina e as políticas governamentais de saúde. Empregando a estrutura dos estudos de história da ciência, tecnologia e medicina, a pesquisa enriquece a historiografia dos hospitais, oferecendo uma visão abrangente de sua importância social durante o período estudado.

Originally, Portuguese hospitals (like their counterparts in other areas of Europe) were conceived as charitable spaces for the poor, sick, vulnerable, orphans, widows, travelers and pilgrims; over time, they evolved into centers for “healing.” This transformation was driven by advances in understanding the etiology of contagious diseases that posed significant threats to public health, such as cholera, plague, typhoid, and yellow fever, but also syphilis and tuberculosis. At the same time, proactive measures, diagnostic tools, treatment modalities, and research techniques played a pivotal role in modernizing medicine, directly influencing the formulation and implementation of various health reforms in which hospitals assumed a prominent role.

Portuguese hospitals were initially administered by the Brotherhood of the Holy Houses of Mercy [Irmandade das Santas Casas da Misericórdia], institutions founded by Queen Leonor (1458-1525), the wife of King João II (1455-1495), in order to perform the 14 works of mercy, which included assisting the sick. Recognizing their charitable mission, the monarchy granted these institutions privileges to oversee various hospitals across the country. The Lisbon House of Mercy was the first to be established, and was tasked with managing the Royal All-Saints Hospital [Hospital Real de Todos-os-Santos]. But unlike the vast majority of hospitals in Portugal prior to that time, the House of Mercy was separated from the Hospital (Portugal, 29 Nov. 1851), which granted the hospital its own administration, making it unique. In 1901, José Curry da Câmara Cabral (1844-1920) published the first general regulations for the São José Hospital and Annexes (Hospital de São José e Annexos), which remained under the direct authority of the Ministry of the Kingdom (Cabral, 1901). After the establishment of the Republic in 1913, the group of hospitals received a new secular name, the Civil Hospitals of Lisbon (Hospitais Civis de Lisboa), granting them autonomy in medical care, administration, and finance (Portugal, 1914).

In this study, we explore the role and evolution of hospitals in the city of Lisbon and how they influenced hospitalization and medical specialization in Portugal, which spans from royal hospitals to civil hospitals, from 1800 (when the royal hospital began operating) to the establishment of the New State [Estado Novo] in 1933 (concluding a political cycle of options for public health). We briefly address the historiography and characterization of the hospital as well as the evolution of these institutions in Lisbon, tracing their development as from the destruction of the first building during the 1755 earthquake. Context is provided for the specialization of medical knowledge, which contributed to the modernization of medicine in Portugal, along with a reflective analysis of the relationship between hospitalization and medical specialization that emphasizes the critical alliance between theory and practice. We conclude with insightful reflections that encapsulate the key findings and implications from our exploration of the historical trajectory of hospitals in Lisbon and their dynamic interplay with medical specialization and public health in Portugal.

By selecting the hospital and the hub of medical specialization in Lisbon as our focal point of study, we approached it from several different perspectives: (1) To assess hospitalization within the framework of hygiene and public health policies, manifested not only through economic strategies but also in the decisions to adapt convents (such as São Lázaro Hospital, Rilhafoles Hospital, Desterro Hospital, Rainha D. Amélia Hospital/Arroios Hospital, Santa Marta Hospital, and Capuchos Hospital) or to create new hospitals modeled on European designs for modern hospital architecture from the nineteenth and twentieth centuries (such as Dona Estefânia Hospital and Rego Hospital). (2) To recognize the hospital’s pivotal role in the emergence of new realms of knowledge within generalist medicine, shaped by experimental or clinical research. This aligns with the tradition of nineteenth-century European schools of scientific research and the innovative concept of “clinic” introduced by Foucault. (3) To explore the hospital as a strategy for professional demarcation and affirmation solidified through educational initiatives either instituted within hospitals or directly associated with them. This includes institutions such as the Royal School of Surgery [Real Escola de Cirurgia], Medical-Surgical School [Escola Médico-Cirúrgica], Medical Faculty of Lisbon [Faculdade de Medicina de Lisboa], and Professional Nursing School [Escola Profissional de Enfermeiros].

The overarching objective is to consider the role of Lisbon’s hospitals based on an analysis of diseases and their control, physicians and medical practices, theories and methodologies, as well as key figures and architectural structures (Démier, Barillé, 2007). To do so, we reexamined existing sources and bibliographies used by various authors in order to reinterpret them through a theoretical lens within the context of the history of science, technology, and medicine, with a particular emphasis on the history of science and medicine (William, 1840; Berridge, 2011; Pickstone, 2000).

In addition to this foundational bibliography, we utilized archival sources related to the history of Lisbon’s hospitals from the National Archives of Torre de Tombo, the libraries of the Lisbon Central and Lisbon North University Hospital Centers, and online digital archives. The Bulletin of São José Hospital and Annexes (Boletim..., 1902, 1903, 1904, 1905, 1906, 1907, 1908, 1909, 1910, 1911, 1912, 1913),^1^ as well as the Statistical Yearbook (Annuario..., 1866, 1890, 1899, 1907b, 1907a, 1908, 1913; Anuário..., 1914, 1922, 1924, 1925, 1926c, 1926b, 1926a, 1927, 1928, 1929, 1930, 1931, 1932, 1933, 1934)^2^ are valuable sources for the study period.

## The hospital: some historiographic reflections

The historiography of the hospital has been a topic of study since the 1960s within the context of Western medicine, with notable contributions from authors such as Florence Nightingale (1863), Erwin Heinz Ackerknecht (1967), Lina Granshaw and Roy Porter (1989; Granshaw, 1992), Hilary Marland (2004), Deborah Brunton (2004), Sophie Chauveau (2011), and Tizian Zumthurm (2020).

The concept of the hospital has evolved over time. The word originates from the Latin word *hospes* [guest/visitor] and transformed into *hospitale* (*domus*) in the thirteenth century, to describe a place where hospitality was practiced by providing accommodation for guests (including travelers or pilgrims) in a setting devoid of luxury, wealth, and symbols of power or social standing (Melo, 2022). During this period, hospitals played a charitable social role, focusing on the care of the poorest, the indigent, and the most vulnerable. During this era, medical interventions carried significant risks, and knowledge of human anatomy and asepsis was rudimentary. Surgeons were often limited to the simplest procedures, and the etiology of infectious diseases, many responsible for widespread and devastating epidemics, was poorly and incompletely understood, while institutions frequently lacked resources for innovation.

The concept of the modern hospital, often referred to as “laboratory medicine,” gained prominence in the period following the French Revolution (Brunton, 2004) at the turn of the nineteenth century, when it became more firmly established. Notable figures such as Xavier Bichat (1771-1802), Phillipe Pinel (1745-1826), René Laennec (1781-1826), Claude Bernard (1813-1878), and Louis Pasteur (1822-1895) emerged as important references in European medicine, including Portugal. At that time, the hospital underwent medicalization (Boulle, 1982), took on a central role in patient empowerment (Ackerknecht, 1967), and laboratory medicine carved out new territories in medical knowledge and practice (Marland, 2004) to mark the “birth of the clinic” (Foucault, 1963). The shift from medicine at the patient’s bedside to laboratory medicine signaled an evolution towards measurable scientific objectivity. This transformation involved the use of statistics^3^ to establish connections between the “big” numbers derived from analyzing patients, diseases and pathogens, and medical successes (Weisz, 2003), fostering novel approaches to address certain pathologies.

The intricate correlation between clinical and pathological evidence was only possible within a hospital environment because of the scale of samples and resources required to endorse a comprehensive, multidisciplinary approach to medicine. The evolving concepts of disease, now understood to be dynamic processes, played a pivotal role in the daily routines of surgeons and clinicians. The hospital became a laboratory in which detailed accounts of patients’ clinical progress became a valuable instrument for advancing scientific understanding of pathologies and discovering effective alternatives to control them.

The nineteenth-century hospital thus served as a tool for social control of national welfare policy, in which patients were subjected not only to clinical examinations but also to autopsies if they did not survive, to acquire causal knowledge about illness post-mortem, granting anatomy a significant role in the advancement of surgery and clinical practice as complementary areas of specialization. Meanwhile, laboratory medicine expanded the scope of medical interventions, spurring the development of medical specialties to obtain a more nuanced understanding of the human body via its various individualized entities, such as organs and systems.

The hospital, as this quintessential setting, played a crucial role in on-site verification of theoretical narratives and assimilation of the new scientific and technical discoveries of the nineteenth and twentieth centuries into the field of medicine. During the 1900s, the medical landscape shifted toward specialization, driven by the professionalization and social recognition of medicine and its practitioners (Brunton, 2004; Veloso, 2017c; Amaral, 2021). This specialization fostered new relationships between spaces, actors, and knowledge, with the hospital prominently positioned within this evolving framework.

As a result, there was a growing focus on cultivating a material and scientific culture that strengthened the role of the medical profession in interventions and oversight of popular health. The influence of specialization is clearly visible in the emergence of new medicalized spaces (Boulle, 1982) that were adapted, replaced, or constructed based on the political leanings and priorities of each regime with regard to their public health agendas.

The historiography of Portuguese hospitals has garnered significant attention from historians. An example is the 2022 collection *The Portuguese Hospitals: from the Middle Ages until today*,^4^ edited by Marta Lobo de Araújo (2022). Additional and noteworthy contributions have been made by Laurinda Abreu, who focuses on the political and social dynamics of poverty and assistance in Portugal from the sixteenth to the eighteenth centuries, emphasizing the significance of the Houses of Mercy (Sá, Lopes, 2008; Abreu, 2015, 2020). Other scholars like Maria Antónia Lopes explore similar themes, delving into the challenges of medical assistance for marginalized populations from the eighteenth to twentieth centuries, with a specific focus on hospitals in Coimbra (Lopes, 2000), Coruche (Correia, 2016) and Alentejo (Silva, 2017). Alexandra Esteves (2021; Esteves et al., 2021) contributes to the social history of health and epidemics, concentrating on the north of the country. Rita Garnel (2011, 2013) and Jorge Alves and Marinha Carneiro (2014) also explore aspects including public health policies, adding to the comprehensive body of work by historians in this field.

Other interdisciplinary publications, primarily authored by medical professionals, also captured our attention. Two notable examples are the compilation of texts by José Leone (1992), published by the Organizing Committee of the 5th Centenary of the Founding of the Royal All-Saints Hospital in 1992 and the 2012 collection *Omnia sanctorum: stories of the history of the Royal All-Saints Hospital and its successors*, edited by Jorge Penedo (2012). Luiz Damas Mora’s *The spirit of the civil hospitals of Lisbon*, in its second edition published in 2013 (Mora, 2013), also offers valuable insights.

Other important contributions to this field include the comprehensive history of Portuguese medicine in the twentieth century entitled *Physicians and society: for a history of medicine in Portugal in the 20th century*, edited by António Barros Veloso (Veloso, Mora, Leitão, 2017), and the 2023 publication edited by Francisco George (2023), *Public health in Portugal: from the 19th century to the new millenium*.

## Hospitals in the city of Lisbon

After the devastation caused by the 1755 earthquake, the Royal All-Saints Hospital in the lower city was demolished and replaced by the Royal São José Hospital on Sant’Anna Hill (Matoso, 2012). The principles of Enlightenment science, championed by figures like Ribeiro Sanches, significantly influenced the political decision not to rebuild the ruined hospital but rather relocate it to a more spacious and better ventilated area, seen as the only way to protect the entire population. In his *Treatise on the conservation of peoples*, Sanches (1756), proposed a series of principles that he believed should guide rulers and decision-makers in collective health emphasizing every state’s imperative to establish laws and regulations to be shield from various diseases and uphold the health of its citizens. He argued that without such measures, even the most knowledgeable and experienced doctors and surgeons would struggle to address an epidemic or any other illness in a city with contaminated air and waterlogged land. A healthy diet and expert medical knowledge, Sanches asserted, would prove futile without first rectifying the malevolence of the atmosphere and preventing further harm. Only magistrates, army generals, and naval and war captains were deemed capable of rectifying devastation under their jurisdiction from such occurrences by enforcing established law (Sanches, 1756).^5^ As a result, a more “airy” and elevated location was selected, adjacent to the dilapidated royal hospital on Sant’Anna Hill. This was the former location of the Jesuit College of Santo Antão-o-Novo, left vacant after the expulsion of the Jesuit Order by the Marquis of Pombal in 1767. In honor of the reigning monarch, José I (1750-1779), the hospital was renamed the Royal São José Hospital [Real Hospital de São José] (Leone, 1992), and served as the foundation for initiatives to provide medical care for the capital and the country; this in turn led to gradual utilization of various other spaces, predominantly former convents. These spaces extended from Sant’Anna Hill outward, throughout the city, farther from its center.

Between 1769 and 1800, the premises of the College of Santo Antão were adapted, not only because of damage from the earthquake but also because the Order considered the organization of this space poorly suited for establishing the city’s central hospital. Despite these changes, the founding principle of the Royal All-Saints Hospital remained unaltered as it was relocated to this former Jesuit school. King João II envisioned the creation of a “great hospital” to aid the poor and sick, which would consolidate the resources of several smaller hospitals in the city, similar to King João V’s Renaissance hospital.

The hospital was open around the clock and housed seven hundred patients in twenty wards, 12 for men (five for medicine and seven for surgery) and eight for women (four for medicine and four for surgery). For men, there were separate wards for treatment of fevers, lepers, sprains, incurables, and the insane (Silva, 1853, p.42). For women, there were four infirmaries for treating fevers and one for incurable diseases (Matoso, 2012, p.65). The multiple wards for treatment of patients with “fevers,” a non-specific symptom and not a pathology, illustrates how little was known from a scientific point of view for precise classification of diseases, not only in Portugal but also across Europe. France offers a well-known case in the controversy between François Broussais (1772-1838) and Pierre Louis (1787-1872), a milestone of modern epidemiology (Morabia, 2004).

As the population grew in the capital, the number of patients expanded to the point that the Royal São José Hospital was unable to meet the swelling demand. Resorting to other available locations in the city became inevitable.

The spaces and assets vacated by the majority of religious congregations after the May 28, 1834, decree signed by Joaquim António de Aguiar (1792-1874) were repurposed to facilitate the expansion of the royal hospital. From 1844 to 1928, the São José Hospital absorbed convents, adapted existing structures, and erected new buildings in close proximity to address persistent overcrowding, an issue consistently highlighted in reports from successive administrators. Additional hospital spaces were occupied in two distinct phases. The first phase, on Sant’Anna Hill, took place between 1844 and 1848 and resulted in the hospital designated the Royal São José Hospital and Annexes. This nomenclature reflected the size of each facility and underscored the joint management of these resources by the Houses of Mercy of Lisbon. The second phase, managed directly by the Royal São José Hospital, began in 1857 and involved extending the hospital’s geographical reach to other parts of the city while simultaneously broadening its sphere of influence and presence in the urban landscape.

The first space that attracted the attention of the Royal São José Hospital’s administration was the convent of São Lázaro, which had always functioned as a lazaretto since its founding in the fourteenth century. By 1837, it was in such disrepair that the costs to maintain it exceeded the funds spent on the sixty patients it housed (Martins, 2012, p.73). It was consequently stripped of its administrative independence and annexed to the royal hospital, and cared for patients with leprosy and other skin diseases until 1918, when they were transferred to the Rego Hospital [Hospital do Rego] and the then Rovisco Pais Colony-Hospital [Hospital-Colónia Rovisco Pais], built in Tocha (Coimbra) in 1947.

In 1848, a medical inspection of the royal hospital was conducted at the request of the Duke of Saldanha (João Carlos Saldanha de Oliveira e Daun) (1790-1876), Minister of the Kingdom, and brought to the attention of Queen Maria II the unsuitable and unhealthy conditions in which patients (particularly women) were accommodated. In response, the Crown sought an alternative convent to relocate these patients from the royal hospital (Sena, 2003). The imperative was to remove the mentally ill from general hospitals due to the challenges of controlling their “unruly” and occasionally violent behavior. The site selected was the convent building that had housed the Rilhafoles (Recolhidas da Encarnação e Carmo), who were subsequently transferred to a different location. The regulations governing the operation of this asylum for the mentally ill were established on April 7, 1851; its management had its own funding and was overseen by the resident medical director. Both indigent and incurable men and women were admitted to this asylum hospital at no cost, while non-indigents and military personnel were charged for services (Silva, 1853). The establishment of this hospital brought with it not only improved (though not yet optimal) conditions for patients, but also the inception of psychiatry as a new medical specialty (Santos, 2011).^6^ Psychiatry was formally recognized by the Portuguese Medical Association, which was founded in 1938 (Portugal, 24 nov. 1938).

In 1911, the institution’s name was changed to Miguel Bombarda Hospital (Hospital Miguel Bombarda) in homage to one of its directors, who played a pivotal role in the modernization of Portuguese medicine, a transformation solidified during the Republican period. Under his directorship, a histology office dedicated to scientific research was established within the hospital. This initiative attracted Marck Athias, who founded the first school of medical research in Portugal (Amaral, 2006).

In 1857, the Convent of Nossa Senhora do Desterro (which was established in 1591 and affiliated with the Cistercian Order) was annexed by the Royal São José Hospital. The convent, which had previously been used by various institutions such as the Navy Hospital and the royal Casa Pia of Lisbon to house orphans and abandoned children, was repurposed to accommodate victims of the yellow fever outbreak that had plagued the city with high mortality since 1856. Despite the unknown nature of the disease, the Medical-Surgical School promptly mobilized to conduct its first histopathological and microscopic pathological anatomy studies at this location, transforming it into a “practical school” for both national and international medical researchers (Mora, 2012).

This laid the groundwork for fundamental research with medical applications, exemplified by the “1911 generation” (Amaral, 2011). In 1862, the hospital admitted the first prostitutes, who had previously been treated at the São José Hospital and Rilhafoles. The institution’s social vocation soon shifted as it specialized in venereology and syphilography, enriched by contributions from medical teaching and training, clinical research, and specialized patient care.

In 1877, the Dona Estefânia Hospital was added to the group, and was the only hospital built from the ground up and was not originally a convent. It was created at the behest of Princess Estefânia, consort of King Pedro V. While visiting the São José Hospital, she was profoundly moved by the impoverished and neglected state of the children. She not only donated her dowry to establish a children’s ward in this hospital but also expressed her desire to build a specialized hospital for their treatment and care (Ferreira, 2023).

Regrettably, neither the princess nor her husband lived to see part of the royal estate of Bemposta carefully chosen for her hospital – an “airy hillside on the outskirts of the city” –, as both succumbed to infectious diseases for which there was no cure at the time. The hospital was inaugurated on July 17, 1877, coinciding with the eighteenth anniversary of her death. The inauguration ceremony was presided over by King Luís and Queen Maria Pia, and the hospital was named in honor of the princess.

The hospital’s design was inspired by the principles advocated by Florence Nightingale, the pioneer of modern nursing and a leading voice in nineteenth-century hospital architecture. It included clean, airy, and well-lit structures built with suitable materials and spatial arrangements, designated separation zones between patient beds, and effective ventilation (Nightingale, 1863). Garden areas and water sources were implemented for water needs as well as leisure, for comfort during convalescence, and an ethic of care was cultivated between health professionals and children.

This hospital served various roles over time, as a backup facility during epidemics and providing essential services for the São José Hospital and the Civil Hospitals of Lisbon, but it solidified its primary function as a pediatric hospital with various clinical and surgical subspecialties while simultaneously offering maternity care. All in all, it became the “laboratory” for the evolution of pediatrics, establishing itself as a prominent maternal and children’s hospital in Lisbon and the country since 1860.

In 1892, the convent of the nuns of the Order of the Conception in Arroios was chosen to house the poor and beggars with contagious diseases, or to temporarily quarantine the sick during epidemic outbreaks that threatened the city. From 1898 onward, the facility also received tuberculosis patients under the patronage of Queen Amélia. This was the first step in royal involvement in the fight against tuberculosis in Portugal, and eventually led to the establishment of the National Tuberculosis Association [Associação Nacional de Tuberculose, ANT] (Vieira, 2016; Veloso, 2017a). It was at this hospital (named Queen Dona Amélia Hospital) that Luiz da Câmara Pestana (1863-1899) died of bubonic plague, which he contracted during the 1899 epidemic in Porto (Leone, 1992, p.85).

**Figure 1:**
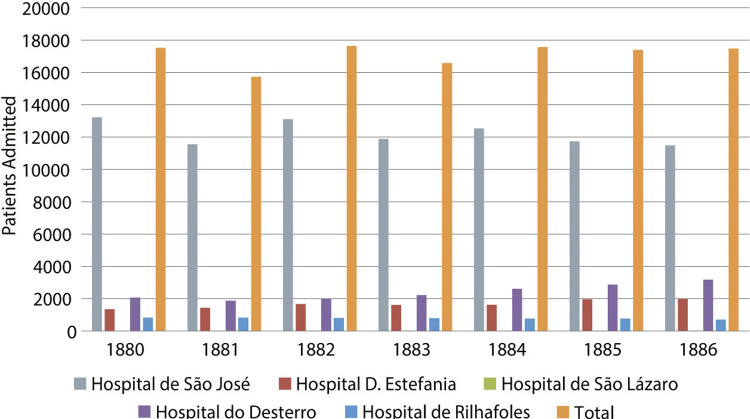
: Patients admitted to the São José Hospital and Annexes (1880-1886) (Annuario..., 1890, 1886)

**Figure 2:**
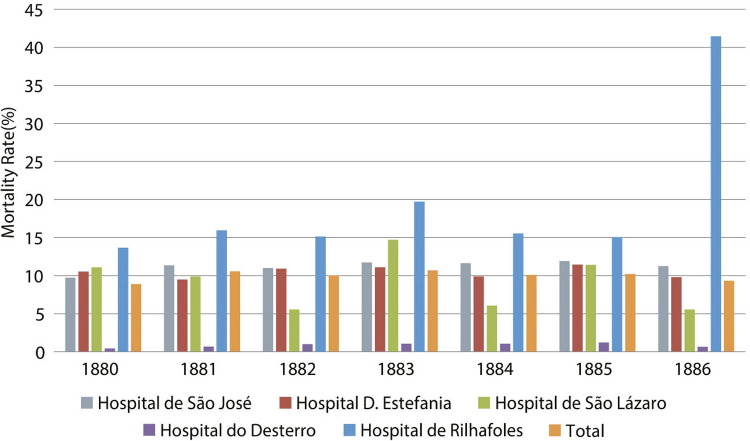
: Mortality rates at the São José Hospital and Annexes (1880-1886) (Annuario..., 1890, 1886)

When we compare the number of patients admitted to various hospitals within the São José Hospital cluster with the corresponding mortality rates (Figures 1 and 2), it becomes apparent that the mortality rates at the two largest hospitals (São José and Estefânia) are similar. This is because an increase in the number of contacts naturally facilitated the spread of contagion, for adults as well as children.

The high mortality rate at Rilhafoles Hospital and São Lázaro Hospital (Figure 1) are noteworthy for distinct reasons. At both institutions, stigmatization, lack of knowledge, and inadequate treatment or even unknown conditions contributed to these figures, and could only be rectified in the latter half of the twentieth century.

Between 1901 and 1910, Curry Cabral conceived and implemented major hospital reform in the city of Lisbon, with political support from Hintze Ribeiro (1849-1907), the Minister of the Kingdom (Veloso, 2017b). He modernized existing hospitals and expanded the complex with two additional units, both isolation hospitals for infectious diseases: Santa Marta Hospital, in 1903, and Rego Hospital, inaugurated in 1906.

In 1903, after the death of the last nun from the Order of Friars Minor living in the Santa Marta de Jesus Convent, this space was adapted to become an isolation hospital for non-febrile infectious diseases (such as syphilis and other venereal diseases, as well as skin diseases). The challenges associated with remodeling the convent were so costly that the planned maternity ward (an integral part of Curry Cabral’s reform) could not be constructed. The hospital was named after Hintze Ribeiro as a tribute to the minister who consistently supported Curry Cabral’s proposals.

In 1906, the 15th International Congress of Medicine was held in Lisbon, bringing together the national and international medical elite. The congress not only marked the inauguration of the new building for Lisbon’s medical-surgical school on Sant’Anna Hill, but also included a visit to the recently inaugurated Rego Hospital, an exemplary model of modern hospital architecture (Pina, Nolasco, 2012), in its social and cultural activities. This hospital was situated in Rego, on the grounds of the Servitas Convent of Our Lady of Sorrows and was designed to be an isolation hospital for febrile infectious and contagious diseases.

Situated far from the city center in an “airy location,” the hospital was built according to the pavilion model of the Eppendorf Hospital in Hamburg, inaugurated in 1889. Comprising 24 independent buildings scattered across a seven-hectare site, it adhered to the hygiene requirements established in the International Sanitary Conferences. The hospital had the capacity to accommodate 516 patients, distributed across wards with thirty, 15, and six beds (Cabral, 1915, p.297-298). This architectural structure concentrated the focus of the hospital by decentralization, considering germ theory as a way to contain and prevent the spread of infectious diseases, as in Europe and the United States (Kisacky, 2017). Modern medicine made the hospital the epicenter for hygienist solutions that drove a new way of controlling disease in the context of public health and is illustrated in the design of the Rego Hospital.

In 1913, this cluster of hospitals was secularly designated as Civil Hospitals of Lisbon, along with the Hintze Ribeiro Hospital (Santa Marta) and the Dona Amélia Hospital (Arroios), but did not forsake the traditions of the past in pursuing the desired modernization of medicine and social recognition for its practitioners.

Finally, the Santo António dos Capuchos Hospital, located near the São José Hospital, was also included in the group after the existing beggars asylum was closed in 1928. With the annexation of this hospital, the cluster of seven hospitals that constituted the Civil Hospitals of Lisbon was complete. Various clinical and surgical services from the São José, Arroios, Desterro, and Dona Estefânia hospitals were set up here, firmly establishing specializations in ophthalmology, otorhinolaryngology, stomatology, and dermatology, with the support of a clinical analysis laboratory and radiology. In 1930, the professional nursing school, named after its founder, Artur Ravarra (1873-1937), was installed in a new building constructed on the hospital grounds and remained here until the turn of the millennium (Matoso, 2012).

During our study period, from 1800 to 1933, a series of reforms took place in the field of charity and public assistance, led by the Monarchy and the Republic. The monarchical period gradually supported the transition from a charitable public hospital service to an assistance system, which was only consolidated in the Republican period. Curry Cabral, the chief nurse of the São José Hospital and Annexes/Civil Hospitals of Lisbon, was most notable during this transition and stated that “at this current time in our civilization, the hospital has ceased to be a simple shelter for patients to also become a practical school where medicine is learned and practiced, and where teachings emerge for those who do not have access” (Cabral, 1915, p.209).

He outlined various strategies to address the overcrowding of poor patients in Lisbon’s hospitals, decentralizing the functions of the central hospital, and rectifying admissions of patients who presented poverty certificates (which were often fraudulent) from all over the country^7^ and consequently overloaded hospitals in the capital. He also created outpatient clinics in various hospitals in the network; although this did not alleviate the central hospital admissions to the expected extent, it attracted other types of patients beyond the poor, indigent, and marginalized. In 1898, a clinic was opened for throat and laryngeal diseases at the Dona Estefânia Hospital, followed by another for syphilitic patients at the Desterro Hospital in 1900, and 12 more clinics in the subsequent year. In 1902, a clinical analysis laboratory was opened, equipped with a photography and microphotography section, microscopes, and equipment for radiography, radiology, and electromagnetism. This laboratory laid the groundwork to train several physicians who began their scientific research here in fields such as pathological anatomy and bacteriology, leading to the emergence of various specialties, which were developed in external institutions such as the Royal Bacteriological Institute and the Tropical Medicine School of Lisbon (Lourenço, Neto, 2011; Amaral, 2008, 2017). The professional nursing school was founded, giving these caregivers a professional identity and the opportunity to progress in hospital and non-hospital careers. A medical statistics department for the hospitals was also created, making it possible to record observations that previously had often died with their authors. This resulted in the publication of the *Bulletin of São José Hospital and Annexes*, starting in 1902. The two hospitals Curry Cabral built in Rego and Santa Marta, which he considered a great advance in hospitalization, could be used during epidemics, avoiding the need for temporary facilities that consumed public funds; they also served as a model for public and urban hygiene, prophylaxis, sanitary police, and public assistance, with the advantage of sporadically sequestering “dangerous” patients (Cabral, 1915, p.231).

Throughout the history of São José Hospital and its annexes, grouping patients by pathologies has always indicated a trajectory of specialization, along with the use of criteria such as specific diseases or target populations (such as tuberculosis, smallpox, leprosy, and syphilis, or children, pregnant women, and the mentally ill). This separation made it possible to separate the work of the state in charitable institutions from care. The Portuguese statistical yearbooks also document this separation. Between 1880 and 1908, data on hospital activity is found in the chapter entitled “Public Assistance,” while from 1909 to 1917 a distinction is made between assistance and public health in order to include the Maritime Disinfection Post, the lazaretto, and the Lisbon Health Station. And from 1918 onward, the hospital data are included under the heading of “Public Health.” These elements indicate the importance of hospitals in constructing an ideal of modernization, which moved from charity to assistance, to culminate in public health as a whole, in a broader approach to diseases and their place in hospitals and in increasingly specialized institutions, such as the Câmara Pestana Bacteriological Institute, the Portuguese Institute of Oncology, ophthalmological care, national assistance to tuberculosis patients (sanatoriums and dispensaries), the Social Hygiene dispensary, and clinics to treat rabies or diphtheria (Anuário..., 1934, p.514-518).

Hospital specialization by disease was also a way to minimize contagion (Sanglard, Costa, 2004). This facilitated the establishment of medical and nursing education institutions closely linked to the hospital, for example, the Royal School of Surgery founded in 1825, and the Medical-Surgical School in 1836 at the São José Hospital and Santa Marta Hospital after the establishment of the Republic. There were also the Higher School of Nursing at the São Lázaro Hospital and the Magalhães Maternity, at Dona Estefânia and Capuchos hospital, respectively. Curry Cabral (1915, p.187) said: “Their most valuable title lies in having served and continuing to serve to train this brilliant array of specialists who today bring luster to the professional practice of Portuguese medicine, especially within the clinical context of hospitals.”

## Final considerations

The hospitals of the nineteenth century mark a crucial shift from the “art of healing” rooted in concepts of well-being and charity to interventionist social assistance. This policy empowered the state, especially in Europe, bestowing not only authority and supervision over patients but also a more intense obligation to validate and recognize the medical profession and medicine. At the same time, science and technology were leveraged during this period to strengthen the dominance of scientific medicine, subsequently facilitating the gradual emergence of medical specialization.

Medical specialization in hospital settings became imperative during the nineteenth century for a variety of reasons. On the one hand, significant demographic growth and migration from rural to urban areas demanded rapid medical intervention to contain epidemics that primarily affected the most vulnerable people (Esteves, 2021). Meanwhile, scientific and technological innovations paved the way toward a new understanding of diseases and epidemics. Etiological disease theories, instruments, methods, and new therapeutic approaches formed the basis of a new scientific tradition in medicine grounded in laboratory research (Amaral, 2006, 2021). Statistics applied to medicine also facilitated the implementation of hygiene reforms, granting medicine and the medical community a hegemonic role in health and disease management (Santos, 2016, 2018).

The specialization of science and medicine led to a new understanding of hospitalization and the role of hospitals in the nineteenth and twentieth centuries, which was particularly influential in the hospital reform led by Curry Cabral in Lisbon.

The character of hospitalization compared to past eras changed, became very different. Hygiene inundated this field and became the essential condition in treating diseases, and its processes became indispensable to implementing prophylaxis. The principles of nosocomial hygiene became preponderant and absolute ... its practice is difficult, and its processes require special techniques (Cabral, 1915, p.173).

Curry Cabral, in leading the organization of the central health care plan in Lisbon in 1901, initiated the first significant structural reform of the Royal São José Hospital. This reorganization marked a new phase defined by structure and action, guided by the central government’s efforts to adapt to scientific and social hygiene requirements as well as administrative and economic conditions in the capital and its population. The medical profession embraced its social role, and as a result of this regulation, the range of services, care, and medical training at the São José Hospital expanded.

In 1918, via Decree n.4,563 (Portugal, 12 jul. 1918) the Civil Hospitals were formally classified and medical specialties emerged. Their services (clinical or surgical specialization units) were legally recognized by the Ministry of the Interior as specialized hospitals, each in a specific area. The São José Hospital became a general polyclinic hospital with 24-hour emergency service, comparable to Hôtel-Dieu Hospital in Paris (Coury, 1967) or St. Bartholomew’s Hospital in London (Power, Waring, 1923). São Lázaro Hospital, where the nursing school operated, had an attached polyclinic service. Desterro Hospital specialized in dermatology, syphilis, and venereology; Dona Estefânia was a general polyclinic hospital for women and children; Arroios and Rego hospitals specialized in infectious diseases; Santa Marta Hospital was a medical training center administered by the medical school of Lisbon; and Miguel Bombarda Hospital specialized in psychiatry.

The history of the Civil Hospitals of Lisbon is closely linked to the prestige of the medical profession and the need for professional affirmation. The São José Hospital was dominated by the surgical-medical tradition. The establishment of the Royal School of Surgery in 1825 led to the formation of the Medical-Surgical School of Lisbon and Porto in 1836, which competed with the country’s only medical school, the Faculty of Medicine, located in Coimbra. The medical career in civil hospitals was very demanding. Medical assistants held the highest position in the technical hierarchy and were the only ones with a government contract; others received a modest stipend. The hospital primarily served as a training ground for doctors to subsequently practice in the private sector, and for this reason, only a select few, the very best physicians, could pursue a career within the hospital.

Experimental medicine gradually replaced book-based medicine in the early twentieth century through investment in small laboratories in hospitals, where charismatic doctors gathered disciples and established research programs in scientific areas to support medicine (such as physiology, histology, microbiology, or parasitology). These training areas were organized into research institutes that played a significant role in modernizing Portuguese medicine in the Faculty of Medicine of Lisbon and Porto, established in 1911 (Amaral, 2011). Hygienic and Pasteurian ideas deeply influenced the medical class, which took center stage in medical specialization. For these doctors, science and technology were indispensable tools in discovering the etiology and therapies that led to the eradication of diseases such as diphtheria, rabies, tuberculosis, and syphilis.

Sexuality, syphilis, and associated venereal diseases had a prominent place in the arguments and programs of social hygiene, not only in Portugal but also throughout Europe, the United States, and Brazil, combining health and morality. The ideal of modernization driven in the name of hygiene and, above all, morality ultimately stigmatized hospitals as being invested by beggars and best avoided by the healthy population.

The Desterro Hospital had wards for men and for women, some of which were controlled by health authorities, while others operated as annexes and support for the São José Hospital. This hospital consequently was a place to where all the excluded members of society converged, along with their pustules, marks, and contamination stigmas. After 1897, thanks to the efforts of Thomaz de Mello Breyner (1866-1933), outpatient consultations for syphilis and venereal diseases were also offered, where domestic workers, seamstresses, laborers, wet nurses, and other women not under the jurisdiction of health authorities were seen and treated. The personal notes of this physician not only illustrate this practice but also reveal that these consultations were also performed in the homes of wealthy women (Bastos, Ramalho, 2017). Breyner initiated a program to assist syphilitic patients, through which he obtained exclusive resources for social prophylaxis of this disease. His connection to the royal house provided him with unique opportunities, and his established international scientific relationships allowed him to use Salvarsan in his patients, which was sent directly by Paul Ehrlich (1854-1915). First World War led syphilis to flourish, followed by a significant increase in investments in assistance and scientific research. Breyner’s tradition at Desterro laid the foundation for establishing a specialization in venereology and dermatology, which later expanded to the Capuchos Hospital and resulted in one of the most significant collections of dermatological wax figures, depicting pathologies that remain relevant in this specialty to this day. These valuable assets are part of the national scientific heritage and should be preserved (Bastos 2011; Bastos, Ramalho, 2017).

Medical specialization in the hospitals of Sant’Anna Hill opened the way for the “colonization” of new spaces in the city closely related to the mother hospital, São José Hospital. Some of these specializations profoundly impacted society, driven by scientific policies. For example, the development of dermatology (a specialty that emerged within the hospital and the medical faculty) became possible with the establishment of the hospital as an essential center for the care of prostitutes from across the country and prevent the spread of infections. This had a significant influence on the consolidation of the specialty of dermatology and the development of hygiene programs during the New State. Similarly, treatment and care of patients with leprosy and mental health issues were crucial undertakings that required specialized solutions. These medical specialties played a vital role in shaping public health policies and addressing specific health challenges faced by society.

In Portugal, as in the rest of Europe, hospitals became vital centers for clinical and pathological training and propelled the advancement of “scientific” medicine. These spaces were the cradle of medical specialization, as observed by George Weisz. Within the São José Hospital and its dependencies, specialization evolved from simple “wards” or “laboratory offices” to well-defined and individualized hospital spaces, which were incorporated into the Faculty of Medicine after 1911.

Medical specialization not only played a role in democratizing medical knowledge and practices through the hospitals, but also disseminated pathologically normalizing diagnoses for the behaviors and attitudes of various social groups. This process extended the influence of medical expertise beyond clinical domains, profoundly shaping the perception and understanding of health and disease in Portuguese history during the nineteeth and twentieth centuries.

## NOTES

1 The first issue of the *Boletim Clínico do Hospital de São José e Annexos* was published in January 1902. The data it contained was collected from patient records cards according to international nosological classification standards and prioritized two types of statistical information: illness and cause of death. The nosological tables were divided into general illnesses, diseases of the nervous, circulatory, respiratory, digestive, or genitourinary systems, pregnancy and childbirth-related conditions, skin diseases, childhood diseases, the results of external violence, and poorly defined conditions. The statistics were published every 15 days and included data from autopsies, clinical laboratory analysis, and services attached to some of the hospitals. Given the scope of this work, we opted not to study individually each disease and its evolution, but in the volumes published from 1902 to 1933 we noted that the most frequently reported illnesses were (in descending order): “other diseases,” epidemic (infectious) diseases, pneumonia (tuberculosis), syphilis, rheumatism, and “poorly defined conditions.” These data corroborate our observations herein as well as the data published in the Portuguese statistical yearbooks.

2 Even though we consulted all the yearbooks published by the National Institute of Statistics from 1880 to 1933, we could not include all the available information in this study, since data on hospital movements expanded to an extent that it was no longer possible to individualize data for the civil hospitals of Lisbon. From 1917 onward, only data by district is presented; from 1919, the classification is done by type of disease; and from 1930, data refers to the 264 hospitals that existed in Portugal at that time.

3 Application of the statistics mentioned here refers to their use as a tool to reconsider hospitals as institutions where large-scale data made it possible to develop new strategies to reorganize healthcare services and improve patient care. It later evolved to provide a better understanding of frameworks to classify certain pathologies such as mental disorders and epidemic (infectious) diseases. This is not the same application of clinical and epidemiological research used by Pierre Charles Alexandre Louis to explain “fevers,” in contrast with the approach used by François Broussais in Paris during the early decades of the nineteenth century.

4 Author’s note: In this and other titles and citations from Portuguese, a free translation has been provided.

5 Original version: “Para mostrar a necessidade de cada Estado ter leis e regulamentos para se proteger de muitas doenças e preservar a saúde de seus súditos; Se estes estiverem ausentes, toda a Ciência da Medicina será de pouca utilidade: porque será impossível para Médicos e Cirurgiões, por mais aprendidos e experientes que sejam, curar uma epidemia ou qualquer outra doença em uma cidade onde o ar é corrupto e a terra alagada. Nem uma boa dieta nem o conhecimento mais preciso nessas artes produzirão os efeitos desejados; sem corrigir primeiro a malignidade da atmosfera e prevenir seus danos. Apenas os magistrados, os capitães-generais nos seus exércitos e os capitães de mar e guerra serão aqueles que, por meio da força das leis decretadas, poderão remediar a destruição daqueles sob sua responsabilidade em ocorrências semelhantes.”

6 The literature on the history of madness and psychiatric hospitals in Portugal, like the history of psychiatry in general, is unfortunately too vast to summarize here and beyond the scope of this paper.

7 According to the *Portuguese Statistical Yearbook*, the São José Hospital and Annexes/Civil Hospitals of Lisbon treated most patients, averaging around twenty thousand (from 1880 to 1910); in comparison, the city of Porto, which ranked second, had an average of roughly 9,900 admissions.
